# RSV Disease Burden in Older Adults: An Italian Multiregion Pilot Study of Acute Respiratory Infections in Primary Care Setting, Winter Season 2022–2023

**DOI:** 10.1111/irv.70049

**Published:** 2024-11-26

**Authors:** Sara Bracaloni, Enrica Esposito, Michela Scarpaci, Tommaso Cosci, Beatrice Casini, Federica Chiovelli, Guglielmo Arzilli, Mauro Pistello, Donatella Panatto, Matilde Ogliastro, Daniela Loconsole, Maria Chironna, Caterina Rizzo, RSVComNet Italy

**Affiliations:** ^1^ Department of Translational Research and New Technologies in Medicine and Surgery University of Pisa Pisa Italy; ^2^ Department of Health Sciences University of Genoa Genoa Italy; ^3^ Hygiene Section, Department of Interdisciplinary Medicine University of Bari “A. Moro” Bari Italy

**Keywords:** acute respiratory infections, primary care, respiratory infection surveillance, RSV

## Abstract

**Background:**

Respiratory syncytial virus (RSV) is a major cause of hospital admission in adults over 65, leading to severe complications and death. However, the disease burden in primary care for older adults in Europe is poorly understood. This pilot study aims to test a study protocol for evaluating the clinical burden of RSV in older adults in primary care settings in Italy.

**Methods:**

In the 2022–23 winter season, we designed a study on RSV burden in individuals over 65 with acute respiratory infections (ARIs) in Liguria, Apulia, and Tuscany, Italy. Recruited patients underwent nasopharyngeal swabs for RSV confirmation and provided epidemiological and clinical data. RSV‐positive patients completed follow‐up questionnaires after 14 and 30 days regarding their clinical conditions, healthcare utilization, and socio‐economic impact.

**Results:**

We enrolled 152 patients with ARIs; 33 (21.7%) tested positive for RSV. The median disease duration was 14 days, with 3% hospitalized. Among RSV‐positive patients, 87% received drug treatment, 52% of whom received antibiotics. After diagnosis, 74% required further GP consultations within 2 weeks. Additionally, 48% incurred extra costs. On day 30, 21% reported health complications or deterioration.

**Conclusions:**

Our pilot study highlights the need for an ARIs surveillance system for older adults in primary care. This is crucial for defining vaccination strategies to reduce the disease burden on these patients and the healthcare system. Moreover, these data are essential for assessing costs and parameters for cost‐effectiveness models, facilitating informed decisions in public health planning and resource allocation.

## 1 Background

Lower respiratory tract infections (LRTIs) significantly contribute to morbidity and mortality in older adults across the United States and Europe, accounting for approximately 1.2 million annual deaths in this population [1]. Respiratory syncytial virus (RSV) stands out among LRTIs' pathogens as a leading cause of hospital admissions in older adults [2], usually peaking in winter in temperate regions and alternating annually between genotypes A and B [3].

Adults with RSV often exhibit milder symptoms than children, but severe cases are not rare, particularly among those with comorbidities, such as immunodeficiency or cardiopulmonary diseases. Fragile adults and older adults hospitalized with RSV can experience significant complications, with 10%–31% requiring intensive care and 3%–17% necessitating mechanical ventilation [4]. Furthermore, RSV has been implicated in the exacerbation of pre‐existing cardiovascular conditions in older adults, potentially triggering critical events such as heart failure, myocardial infarction, and stroke [5].

Recent evidence indicates that RSV may account for up to 25% of the excess winter mortality previously ascribed to influenza, with RSV patients experiencing poorer outcomes compared to those with influenza or human metapneumovirus [6, 7, 8]. These results highlighted the substantial socio‐economic impact of RSV on healthcare systems globally, yet the precise disease burden in the general older adults' population, particularly in Italy, remains unknown. This knowledge gap is partly due to limited awareness among healthcare providers and the absence of routine RSV testing [6, 9].

The growing focus on RSV in older adults is supported by the recent authorization of new vaccines available in the market in the United States and Europe [10, 11]. With the advent of these vaccines, understanding the disease burden in people over 60 is crucial for designing effective implementation strategies and highlights the need for comprehensive surveillance at both national and European levels in order also to evaluate implemented strategies [12]. RSV has only recently been included in the list of the European Surveillance System (TESSy) data to be reported to ECDC by EU Countries [13].

However, although some European countries provide of seasonal RSV trends, the absence of a standardized surveillance protocol hampers the comparability of data across countries and limits the assessment of healthcare burdens and the impact of vaccination programs [14].

In Italy, the Italian Influenza Surveillance Network (InfluNet) has been tracking influenza‐like illnesses (ILI) since 2000, with the inclusion of RSV monitoring since the 2022–2023 season; yet a comprehensive understanding of RSV's clinical burden in older adults in primary care is still lacking [15]. Since 2019, as part of the multinational European RSV ComNet Project, we have been investigating the national clinical burden of RSV in the pediatric population within primary care settings [16]. In the winter season 2022–2023, we decided to expand our RSV study protocol to include the over‐65 population, with a pilot study across three Italian regions, aiming to establish a study protocol that addresses the knowledge gap regarding the burden of RSV in older adults in a primary care setting. This is the first pilot study that aims to explore the health and socio‐economic burden of RSV's disease in older adults' Italian population.

## 2 Methods

### 2.1 Study Design and Settings

This pilot study spanned the 2022–2023 winter season, from week 48 of 2022 (November 28^th^ to December 4^th^) to week 13 (March 27^th^ to April 2^nd^) of 2023. It involved a collaborative effort among three centers in three different Italian regions: the University of Genova in Liguria (Northern Italy), the University of Pisa in Tuscany (Central Italy), and the University of Bari in Apulia (Southern Italy). The University of Pisa coordinated the study, overseeing both coordination and data analysis. The enrollment of GPs was promoted through meetings with their representatives to explain the design and purpose of the study in the three cities hosting the University centers (Pisa, Genova, and Bari); subsequently, the GPs' participation was based on their willingness to contribute to the study. A total of 17 General Practitioners (GPs) from three regions participated in the study.

### 2.2 Patient Recruitment and Case Definition

The study included voluntary patients aged 65 years and older, assisted by the participating GPs, who were diagnosed with an acute respiratory infection (ARI) during routine outpatient visits. In cases where patients were not self‐sufficient, their caregivers were approached. Patients with a previous diagnosis of RSV in the current season, limited proficiency in Italian, cognitive impairment, or recent significant family bereavement were excluded. Informed consent was obtained from all participants or their legal guardians. The ARI case definition adhered to the criteria set by the European Centre for Disease Prevention and Control, encompassing a sudden onset of symptoms and at least one of the following four respiratory symptoms: cough, sore throat, shortness of breath, or coryza, coupled with a clinical assessment confirming an infectious etiology [17]. General practitioners had not received specific training for identifying an RSV infection and dealing with a patient infected by it: Study enrollment was offered to any patient who met the definition of ARI case, aged over 65 and without exclusion criteria; the diagnostic and therapeutic course was not influenced by study participation or not. The enrollment criteria for patients' participation were identical to those used by the European RSV ComNet Project, except for the patients' age (> 65 vs. < 5 years old).

### 2.3 Laboratory Methods

The enrolled patients underwent nasopharyngeal swabbing performed by their GPs. The samples were then analyzed in the reference laboratories of the participating centers using Multiplex RT‐PCR (Allplex™ Respiratory Full Panel Assay) testing. This method was employed to detect and identify a comprehensive range of respiratory infectious agents, both viral and bacterial, as detailed in Table 1.

**TABLE 1:** Multiplex RT‐PCR assay for the detection and identification of 26 respiratory pathogens.

RSV	Respiratory syncytial virus A (RSV A) Respiratory syncytial virus B (RSV B)
Influenza virus	Influenza A virus (Flu A) Influenza A‐H1 (Flu A‐H1) Influenza A‐H1pdm09 (Flu A‐H1pdm09) Influenza A‐H3 (Flu A‐H3) Influenza B virus (Flu B)
Other viruses	Bocavirus 1/2/3/4 (HBoV) Coronavirus 229E (229E) Coronavirus NL63 (NL63) Coronavirus OC43 (OC43) Human rhinovirus (HRV) Adenovirus (AdV) Enterovirus (HEV) Metapneumovirus (MPV) Parainfluenza virus 1 (PIV 1) Parainfluenza virus 2 (PIV 2) Parainfluenza virus 3 (PIV 3) Parainfluenza virus 4 (PIV 4)
Bacteria	Bordetella parapertussis (BPP) Bordetella pertussis (BP) Chlamydophila pneumoniae (CP) Haemophilus influenzae (HI) Legionella pneumophila (LP) Mycoplasma pneumoniae (MP) Streptococcus pneumoniae (SP)

### 2.4 Data Collection

Upon enrollment, GPs administered a standardized questionnaire (T0 questionnaire) to each patient to gather essential information, including demographic information, pre‐existing clinical conditions, vaccination status (influenza, pneumococcal, and COVID‐19 vaccinations), symptoms, and recurrent pharmacological treatments before the GP visit. At the same time, the nasopharyngeal swab was performed.

For patients who tested positive for RSV, follow‐up structured telephone interviews were conducted by researchers involved in the project at 14 days (T14) and 30 days (T30) to capture a comprehensive overview of the patient's ongoing clinical condition, medication regimen (only at T14), any further medical consultations or healthcare needs, potential complications (only at T30), and the socio‐economic impact of the infection on their daily lives.

All data collected from T0 questionnaires, follow‐up interviews (T14 and T30) and laboratory samples were stored on a web‐based platform. This is a custom‐built platform that was specifically developed for the study, accordingly to the researchers' requests and with different kinds of accounts and permissions depending on the role played by the account holder (researchers/GPs/laboratory/head of research center). The platform was used by the three regional study groups to standardize data collection and management and support the data quality check.

### 2.5 Statistical Analysis

A descriptive analysis of the study population was conducted to provide a general overview of the characteristics of the individuals included in the study at the different times of observation (T0, T14, T30). We performed linear regression to evaluate how age, chronic cardiovascular and respiratory diseases, and T0 symptoms influenced the duration of illness, healthcare utilization measured as the number of contacts had with the GPs, antibiotic prescription, and occurrence of complications. Data was analyzed using the statistical software R (version 4.2.3) [18].

## 3 Results

### 3.1 Sample Characteristics

In Table 2, all the characteristics of the ARI cases at the time of recruitment are reported by RSV infection status.

**TABLE 2:** Features of the recruited ARI cases at T0.

		Total population	Patients with RSV infection	Patients without RSV infection
		*n* = 152	*n* = 33	*n* = 119
*Demographic and social characteristics*				
**Sex**				
	Men	68 (44.7%)	16 (48.5%)	52 (43.7%)
	Women	84 (55.3%)	17 (51.5%)	67 (56.3%)
**Age category**				
	65‐74	70 (46.0%)	13 (39.4%)	57 (47.9%)
	75‐84	57 (37.5%)	10 (30.3%)	47 (39.5%)
	Over 85	25 (16.5%)	10 (30.3%)	15 (12.6%)
**Geographic region**				
	Pisa	39 (25.6%)	12 (36.4%)	27 (22.7%)
	Genova	86 (56.6%)	16 (48.5%)	70 (58.8%)
	Bari	27 (17.8%)	5 (15.6%)	22 (18.5%)
*Pre‐existing clinical conditions and vaccination status*				
**Cardiovascular diseases**				
	Yes	84 (55.3%)	23 (69.7%)	61 (51.3%)
	No	68 (44.7%)	10 (30.3%)	58 (48.7%)
**Chronic respiratory diseases**				
	Yes	38 (25.0%)	8 (24.2%)	30 (25.2%)
	No	114 (75.0%)	25 (75.8%)	89 (74.8%)
**Diabetes mellitus**				
	Yes	15 (9.9%)	5 (15.2%)	10 (8.4%)
	No	137 (90.1%)	28 (84.8%)	109 (91.6%)
**Flu vaccine**				
	Yes	117 (77.0%)	26 (78.8%)	91 (76.5%)
	No/Data missing	35 (23.0%)	7 (21.2%)	28 (23.5%)
**Pneumococcal vaccine**				
	Yes	66 (43.4%)	13 (39.4%)	53 (44.5%)
	No/Data missing	86 (56.6%)	20 (60.6%)	66 (55.5%)
**COVID‐19 vaccine**				
	Yes	150 (98.7%)	32 (97.0%)	118 (99.2%)
	No	2 (2.3%)	1 (3.0%)	1 (0.8%)
*Laboratory results*				
**Influenza A/H3N1**				
	Yes	16 (10.5%)	1 (3.0%)	15 (12.6%)
	No	136 (89.5%)	32 (97.0%)	104 (87.4%)
**SARS‐CoV 2**				
	Yes	6 (3.9%)	0 (0%)	6 (5.0%)
	No	146 (96.1%)	33 (100%)	113 (95.0%)
**Rhinovirus**				
	Yes	23 (15.1%)	0 (0%)	23 (19.3%)
	No	129 (84.9%)	33 (100%)	96 (80.7%)

#### 3.1.1 Demographic and Social Characteristics of all ARI Recruited Cases

One hundred fifty‐two patients with ARIs were enrolled by the participating GPs during the 2022–2023 winter season. Of these, 33 (21.7%) ARI cases tested positive for RSV. The median age was 78 years (min 66–max 91). In terms of disability, 12.1% (four patients) were not self‐sufficient, and none were institutionalized.

#### 3.1.2 Pre‐Existing Clinical Conditions and Influenza, PCV, and COVID‐19 Vaccination Status of RSV Confirmed Cases

The majority, 81.8% (27/33 patients), presented pre‐existing chronic conditions. Of these, 69.7% (23 patients) had cardiovascular diseases, including hypertension, atrial fibrillation, and heart failure. Chronic respiratory diseases were present in 24.2% (eight patients), and 15.1% (five patients) had diabetes mellitus. The influenza vaccination coverage was 78.8%, whereas pneumococcal vaccine coverage was 39.4% and 96.9% of patients were vaccinated against COVID‐19.

#### 3.1.3 Laboratory Results

Within this RSV‐positive group, two cases (6.1%) were identified as serotype A, whereas the remaining 31 cases (93.9%) were RSV serotype B. Most RSV‐positive patients (81.8%, 27 patients) did not have co‐infections. Coinfections were found in six patients, with Influenza A/H3N1 virus in one case and *Haemophilus influenzae* in five cases.

#### 3.1.4 Access to Primary Care Services

The median time for GP visit from symptom onset was 4 days (min 0, max 19 days). Prior to the GP visit, 15.1% (5 patients) had already started antibiotic treatment.

### 3.2 Trend of ARI and RSV Cases

**FIGURE 1:**
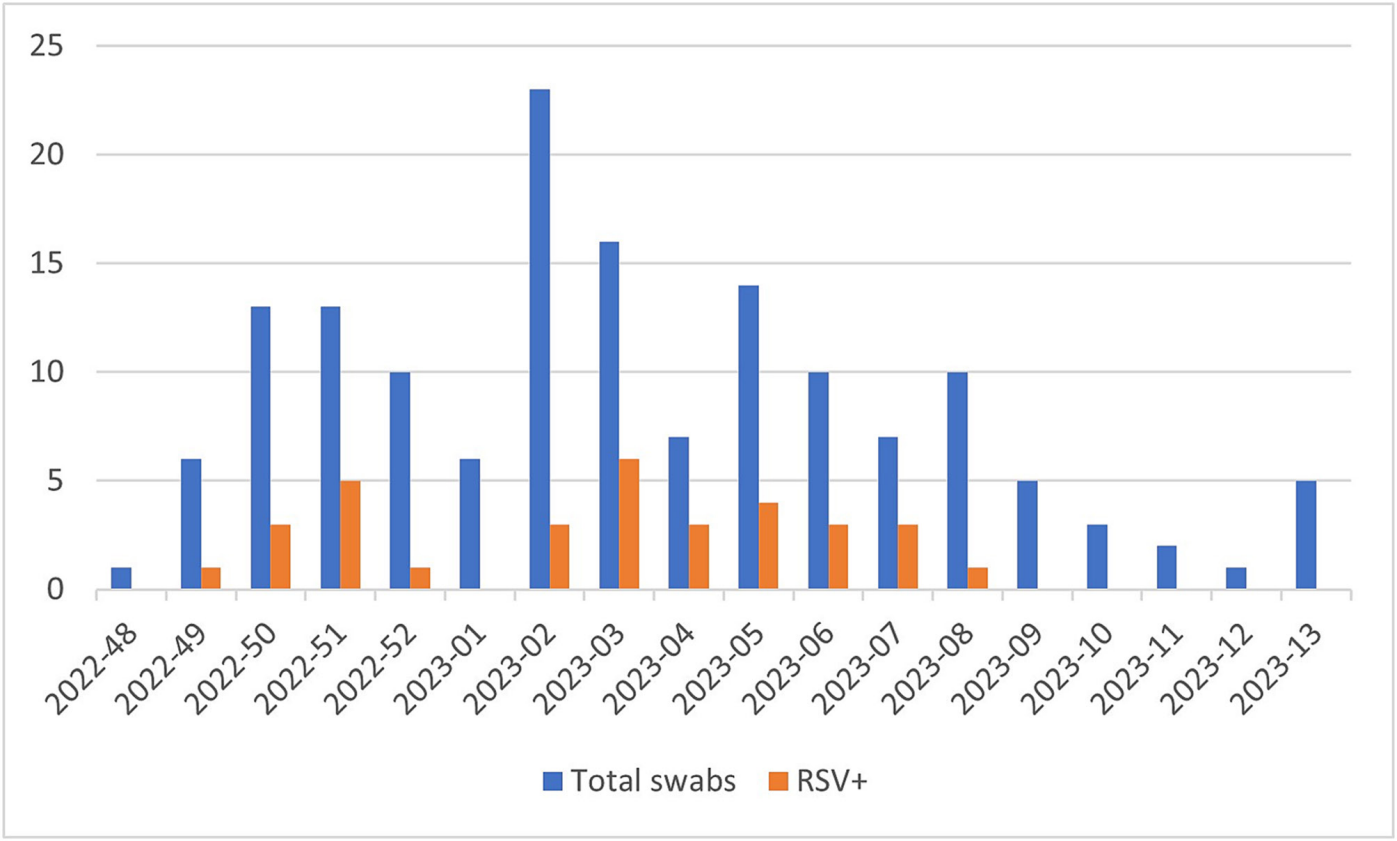
Trend of ARI and RSV cases recruited in the study, winter season 2022–2023. Patients with ARIs were recruited in the study from week 48 of 2022 (November 28^th^ to December 4^th^) to week 13 (March 27^th^ to April 2^nd^) of 2023. The first RSV cases were detected among these in week 49 of 2022. The RSV‐positive patients were concentrated in the latter half of December 2022 and from the second to the eighth week of 2023, with a peak in the third week of 2023.

In Figure 1, the temporal distribution of RSV cases is reported. The first cases were detected from week 49 in Genoa (07/12), followed by Pisa on 19/12 (week 51) and Bari on 10/01 (week 2023‐2). The RSV‐positive patients were concentrated in the latter half of December 2022 and from the second to the eighth week of 2023. The incidence of RSV infections peaked in the third week of 2023.

### 3.3 Follow‐Up and Treatment

Thirty‐one and 29 patients (93.9% and 87.9%, respectively) with RSV infection completed the T14 and T30 follow‐up questionnaires. The median duration of illness was 14 days (range 5–48 days). After diagnosis, 27/31 patients (87.1%) received a drug treatment, of which 18/27 (66.7%) received more than one drug, 16 (59.2%) were prescribed antibiotics, 7 (2.6%) received aerosol therapy, 6 (22.2%) cortisone tablets, and the same percentage used paracetamol. Nonsteroidal anti‐inflammatory drugs were used by 5 patients (18.5%).

#### 3.3.1 Symptoms Persistence and Health System Utilization

At T14, 48.4% (15 patients) still had symptoms, predominantly cough (productive in 19.3%, nonproductive in 25.8%). Other symptoms included shortness of breath (9.6%), coryza or fatigue (6.4%), and sore throat or dehydration or loss of appetite (3.2%), no fever was reported. By T30, 34.5% (10 patients) still had symptoms, mainly non‐productive cough (20.7%), 20.7% (6 patients) reported complications such as pleural effusion, bronchitis, pneumonia, or exacerbation of pre‐existing conditions (Table 2).

Health system utilization included 74% contacting their GPs, 32% needing at least one outpatient visit, and 16% consulting a specialist. Two patients (6%) had emergency room visits access, with one requiring hospitalization.

#### 3.3.2 Complications and Socio‐Economic Impact

The socio‐economic impact was significant, with 48.4% (15 patients) incurring extra costs, primarily for medications, specialist consultations, and laboratory tests. Among the patients, 6.5% (two out of 31) reported work absences due to the infection, and a similar impact was noted on the work of caregivers.

### 3.4 Regression Analysis

The results of the regression analysis showed that productive cough increased antibiotic prescription by 0.49 times at T14 (*p* = 0.005), fever was associated with a 0.42‐times increase in diagnosed complications at T30 (*p* < 0.001), and loss of appetite and sore throat led to an increase in family doctor home visits (0.5, *p* = 0.002) (1, *p* < 0.001). However, fever decreased the number of calls to the family doctor (−2.55, *p* < 0.003) and fatigue reduced the number of outpatient visits (−1.2, *p* = 0.004). Duration of illness was never significantly affected by the confounders considered.

## 4 Discussion

This paper reports the results of the pilot study on RSV clinical and socio‐economic burden in the older adults' population in primary care settings in Italy. We conducted the study by monitoring viral and bacterial infections during the winter season 2022–2023, for a total observation of 18 weeks, in three different Italian regions. The results highlighted all infectious agents responsible for ARIs, among which the most represented was RSV. The first cases were identified from December 7th until more than one in five enrolled patients tested positive for RSV (21.7%), indicating a more considerable impact in this age group than commonly expected, confirmed also by the InfluNet national data [19]. The most represented serotype in our population was serotype B in 93.9% of cases, in line with the existing literature [20]. In addition, our study also showed a number of cases related to other diseases such as Influenza A/H3N1 and *Haemophilus influenzae* .

The ARI symptoms reported at the onset of the disease in our study, such as nasal congestion and cough, align with those frequently described in the literature in older adults for RSV [2] and the other infectious diseases identified. Also, the median duration of the disease was consistent with available literature [21]. Our study highlights another important focus related to the target population considered. As expected, a significant number of patients had chronic conditions such as cardiovascular and respiratory diseases: It becomes more important to stress how these RSV‐positive patients may be exposed to an increased risk of RSV infections severity [5, 22].

Our pilot study also highlighted the treatment prescribed by GPs. The standard care for RSV infection in adults is mostly supportive [23], yet our data suggest a substantial use of antibiotics and corticosteroids besides standard anti‐inflammatory drugs. This could be attributed to the high rate of chronic comorbidities among patients. However, it also aligns with previous research indicating a tendency for antibiotic over prescription in ARIs [24]. Unfortunately, due to the low number, it was impossible to estimate the appropriateness of antibiotic prescriptions.

Our study also had the innovative aspect of assessing the impact of RSV disease on patients' socioeconomic burden. Our data showed that this burden was significant, with nearly half of the patients reporting additional out‐of‐pocket costs. The burden is also extended to caregivers, who might face economic and job‐related challenges due to caregiving demands [25]. Another socioeconomic aspect highlighted is the strain on the primary care system, evidenced by the high frequency of GP consultations post‐diagnosis. These two aspects pave the way to analyze the issue related to the epidemiological trend of RSV without disconnecting it from the socio‐sanitary aspects it entails on the individual patient, their family context, and the system that takes care of them.

Although our study's co‐infection rates support existing literature on the severity and mortality risks associated with bacterial co‐infections in RSV [26], the observed hospitalization rate (3%) cannot be generalized due to the small sample size.

The study had some limitations, including a small sample size and challenges in GP participation, potentially influenced by limited awareness of RSV's significance in adults [6]. The study's late start also missed early seasonal data. Future research should aim for larger sample sizes and earlier data collection. It would be desirable to organize meetings with a greater number of GPs rather than only their representatives, in order to succeed in recruiting a larger share of doctors, given the low participation: We estimate that more than 600 GPs work in the three cities, but only 22 of them decided to participate in the study and actually only 17 actively contributed to the study. Additionally, raising awareness among GPs about the importance of RSV infection in adults is crucial for better participation in future studies. Therefore, the organization of meetings with territorial groups of GPs, before the start of the epidemic season, might be a promising approach.

Data collected, although limited, confirms the seasonality of RSV observed in Italy during the 2022–2023 winter season, showing an earlier peak, between December 2022 and January 2023. Due to the small number of patients recruited, the study was unable to offer detailed analysis on RSV seasonality, except for the week with the highest number of cases: This limitation may be overcome in the future by expanding the sample size.

To our knowledge, this is the first study to report socio‐economic and clinical data on RSV in community‐dwelling older people in Italy. Other studies recently investigated the burden of RSV in adult patients. For example, the study of Narejos‐Pérez et al. [27] assessed the burden of RSV‐ARIs in community‐dwelling European adults (≥ 50 years) in 2019–2020 and 2020–2021 seasons, partially overlapping with the COVID‐19 pandemic. Their results show a significant burden, describing a notable worsening of the patients' quality of life during the first 7 days of the ARI episode, and revealing a remarkable impact on the patients' vitality with worsening fatigue during the first 14 days. Moreover, complications tended to be more frequent in participants with RSV‐ARI than those without (17.4% vs. 3.0%). Still, comparing studies is difficult because of many factors, such as different case definitions, methodology, geography and timing.

Although our study provides limited data, it gives a good indication of what to investigate to learn how this disease, still little considered by the territorial primary care service, is actually an additional burden on a complex system. Therefore, it needs to be recognized as not only a clinical issue, but also as an impact on the life of the individual, the community, and from an economic perspective on the population in which this disease is widespread.

In conclusion, our pilot study is an important step toward the implementation of an ARI surveillance system targeted for older adults and of vaccination strategies that are critical steps toward mitigating the impact of RSV on this population, thereby reducing hospitalizations, healthcare costs, and the overall disease burden especially in the frail older adult's population.

Future research should focus on identifying high‐risk groups for targeted vaccination strategies and understanding the national burden of RSV. This will be crucial in shaping effective vaccination strategies and evaluating the impact of the implemented program.

## 5 Conclusions

The clinical and epidemiological data derived from this study could provide important insight for understanding the burden of RSV in older adults within primary care settings. These results not only emphasize the necessity for enhanced surveillance and vaccination programs with available vaccines, but also reveal the significant economic implications of RSV management.

By integrating these findings into cost‐effectiveness models, policymakers can make evidence‐based decisions that optimize healthcare resources, ensuring both improved patient care and economic sustainability.

## Author Contributions

**Sara Bracaloni:** conceptualization, investigation, data curation, methodology, project administration, writing – original draft, writing – review and editing. **Enrica Esposito:** conceptualization, investigation, data curation, project administration, supervision, validation, writing – original draft, writing – review and editing. **Michela Scarpaci:** conceptualization, investigation, writing – review and editing. **Tommaso Cosci:** conceptualization, investigation, writing – review and editing. **Beatrice Casini:** conceptualization, funding acquisition, investigation, writing – review and editing. **Federica Chiovelli:** investigation, project administration, resources. **Mauro Pistello:** data curation, investigation, project administration. **Guglielmo Arzilli:** conceptualization, formal analysis, methodology, software, writing – original draft, writing – review and editing. **Donatella Panatto:** project administration, resources, supervision, writing – review and editing. **Matilde Ogliastro:** investigation, project administration, resources. **Daniela Loconsole:** conceptualization, resources, supervision, validation, writing – review and editing. **Maria Chironna:** conceptualization, project administration, resources, supervision, writing – review and editing. **Caterina Rizzo:** conceptualization, funding acquisition, investigation, project administration, resources, supervision, writing – review and editing. **RSVComNet Italy Working Group:** funding acquisition, project administration, resources.

## Ethics Statement

The study protocol received approval from the Ethical Committee “Comitato Etico di Area Vasta Nord Ovest (CEAVNO) per la Sperimentazione clinica” of the Tuscany Region, Italy, under protocol number 22871_Dini, dated 25 October 2022. Additionally, all participating sites adhered strictly to their respective local ethical guidelines and procedures throughout the study.

## Conflicts of Interest

CR participated in Advisory Board and Expert scientific discussion for Seqirus, MSD, GlaxoSmithKline (GSK), Sanofi, and AstraZeneca. The other authors declare no conflicts of interest.

### Peer Review

The peer review history for this article is available at https://www.webofscience.com/api/gateway/wos/peer‐review/10.1111/irv.70049.

## RSV ComNet Italy Study Contributors (Group Authorship)

*Hygiene Section, Department of Interdisciplinary Medicine, University of Bari “A. Moro”, Bari, Italy*: Francesca Centrone; *Department of Health Sciences, University of Genoa, Italy*: Giancarlo Icardi, Piero Luigi Lai, Carola Minet, Giada Garzillo, Bianca Roncan, Sara Tardito, Marta Crocetti. *General practitioners*: Serena Batini, Cinzia Benedetti, Lyubov Blokh, Filomena Cetani, Lorena Cocciaro, Manila Notarbartolo, Luca Puccetti, Antonio Zoppi, Tecla Mastronuzzi, Cardini Marco, Carraro Andrea, Farese Antonio, Lombardi Alessandro, Messina Valeria, Pennacchietti Carlotta, Polese Marco, Proietti Carlo.

## Data Availability

Data are available on request due to privacy/ethical restrictions.
